# Secondary Metabolites from Vietnamese Marine Invertebrates with Activity against *Trypanosoma brucei* and *T. cruzi*

**DOI:** 10.3390/molecules19067869

**Published:** 2014-06-11

**Authors:** Nguyen Phuong Thao, Joo Hwan No, Bui Thi Thuy Luyen, Gyongseon Yang, Soo Young Byun, Junghyun Goo, Kyung Tae Kim, Nguyen Xuan Cuong, Nguyen Hoai Nam, Chau Van Minh, Thomas J. Schmidt, Jong Seong Kang, Young Ho Kim

**Affiliations:** 1 College of Pharmacy, Chungnam National University, Daejeon 305-764, Korea; E-Mails: thaonp@imbc.vast.vn (N.P.T.); luyenbthoaduoc@gmail.com (B.T.T.L.); devilkkt@nate.com (K.T.K.); 2 Institute of Marine Biochemistry (IMBC), Vietnam Academy of Science and Technology (VAST), 18-Hoang Quoc Viet, Caugiay, Hanoi 10000, Vietnam; E-Mails: cuongnx@imbc.vast.vn (N.X.C.); namnguyenhoai@imbc.vast.vn (N.H.N.); cvminh@vast.ac.vn (C.V.M.); 3 Chemical Biology of Pathogens Group, Institute Pasteur Korea, Seongnam-si, Gyeonggi-do 463-400, Korea; E-Mails: celestial7@ip-korea.org (J.H.N.); gyongseon@ip-korea.org (G.Y.); runaway1010@ip-korea.org (S.Y.B.); goojh@ip-korea.org (J.G.); 4 Institute of Pharmaceutical Biology and Phytochemistry (IPBP), University of Münster, PharmaCampus, Corrensstrasse 48, Münster D-48149, Germany; E-Mail: thomschm@uni-muenster.de

**Keywords:** soft coral, echinoderm, marine natural product, *Trypanosoma brucei*, *Trypanosoma cruzi*, neglected tropical diseases

## Abstract

Marine-derived natural products from invertebrates comprise an extremely diverse and promising source of the compounds from a wide variety of structural classes. This study describes the discovery of five marine natural products with activity against *Trypanosoma* species by natural product library screening using whole cell *in vitro* assays. We investigated the anti-trypanosomal activity of the extracts from the soft corals and echinoderms living in Vietnamese seas. Of the samples screened, the methanolic extracts of several marine organisms exhibited potent activities against cultures of *Trypanosoma brucei* and *T. cruzi* (EC_50_ < 5.0 μg/mL). Among the compounds isolated from these extracts, laevigatol B (**1**) from *Lobophytum crassum* and *L. laevigatum*, (24*S*)-ergost-4-ene-3-one (**2**) from *Sinularia dissecta*, astropectenol A (**3**) from *Astropecten polyacanthus*, and cholest-8-ene-3*β*,5*α*,6*β*,7*α*-tetraol (**4**) from *Diadema savignyi* showed inhibitory activity against *T*. *brucei* with EC_50_ values ranging from 1.57 ± 0.14 to 14.6 ± 1.36 μM, relative to the positive control, pentamidine (EC_50_ = 0.015 ± 0.003 μM). Laevigatol B (**1**) and 5*α*-cholest-8(14)-ene-3*β*,7*α*-diol (**5**) exhibited also significant inhibitory effects on *T. cruzi*. The cytotoxic activity of the pure compounds on mammalian cells was also assessed and found to be insignificant in all cases. This is the first report on the inhibitory effects of marine organisms collected in Vietnamese seas against *Trypanosoma* species responsible for neglected tropical diseases.

## 1. Introduction

Neglected tropical diseases (NTDs) are life threatening or disabling infections affecting more than a billion people worldwide. People suffering from NTDs hence constitute an unattractive market to private sector research and development investment [1]. This situation is a matter of significant concern-NTDs not only affect health directly, but they also represent a serious socioeconomic impact that perpetuates poverty in a sizable part of the world’s population [2,3]. The drugs that are currently available to treat NTDs exhibiting many toxic effects [4], which makes the search for more effective and safer medications to fight these conditions mandatory. However, a fundamental problem regarding NTDs is to convince pharmaceutical companies to engage in the investigation and development of new, cheap and effective treatments, since the population affected by these diseases cannot afford expensive drugs [3,5]. Currently, seventeen NTDs are defined as such, including three major protozoan diseases: human African trypanosomiasis (HAT, more commonly known as sleeping sickness, caused by *Trypanosoma brucei* subspecies), Chagas’ disease (caused by *T. cruzi*), and leishmaniasis (a group of infections with *Leishmania* parasites including, e.g., kala azar or black fever), which affect many millions of people worldwide, although the number of new infections currently appears to be decreasing [6]. This decrease is certainly an achievement of the consequent strategies of disease management and prevention followed by WHO [7] and may in the future be enhanced by development efforts from academia, private initiatives, and nonprofit organizations [6,8].

Marine environments, particularly in the tropics, have greater species richness than forests. These diverse species are capable of producing a wide variety of the chemical compounds with unique structures and functions, and many of these compounds can be used for the development of novel drugs [9]. Thus, research on the use of marine natural products as pharmaceutical agents has been steadily increasing [10]. Secondary metabolites produced by diverse marine organisms thus represent a huge repository of the chemical structures for searching new drugs and which can contribute to improved public health conditions in tropical developing countries [11,12].

**Figure 1:**
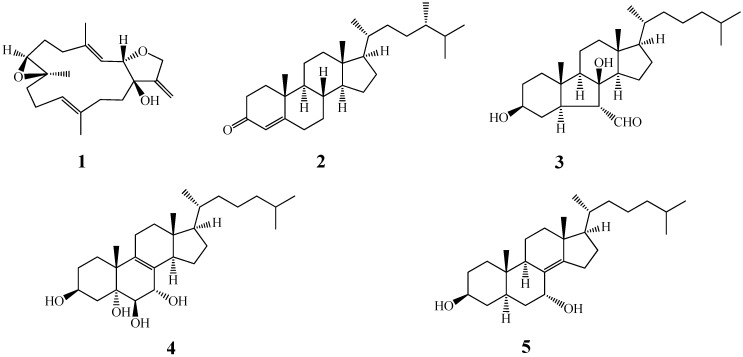
The chemical structures of compounds **1**–**5**.

As a part of our current screening for biologically active compounds from marine organisms against NTDs [13], we found that the methanolic extracts of the soft corals and echinoderms showed potent inhibitory activities against *T. brucei* and *T. cruzi* (EC_50_ < 5.0 μg/mL) with no toxicity on the mammalian HEK293T (human embryonic kidney) and HepG2 (human hepatoma) cell lines. We herein report on the anti-trypanosomal activity of the major pure constituents from five of the most active extracts against the mentioned *Trypanosoma* species. The respective inhibitory effects as well as their potential cytotoxicity were investigated. Our results show that these compounds **1**–**5** (Figure 1) are active against *T.* *brucei* and *T. cruzi* and do not have any significant cytotoxic effects.

## 2. Results and Discussion

### 2.1. High Throughput Screening of Natural Product Library

**Figure 2:**
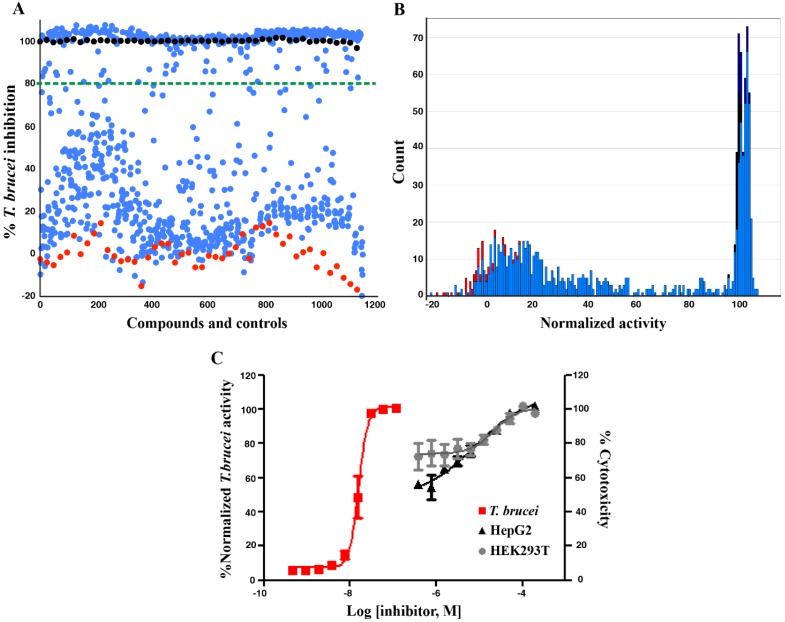
High throughput screening against *T.* *brucei* growth: (**A**) Distribution plot of the 861 natural compounds and extracts (blue), 0.5% DMSO (red), and pentamidine at EC_100_ (black); (**B**) Frequency distribution of the 861 natural compounds and extracts based on binned normalized activity; (**C**) Dose response curve of pentamidine against *T. brucei*, HEK293T, and HepG2 cells.

To identify inhibitors of *Trypanosoma* *brucei* growth, we conducted resazurin-based high throughput screening of a proprietary library consisting of 861 purified natural products and sub-fractionated extracts (including 433 extracts and 428 pure compounds). The library was composed of both fractionated single components as well as sub-fractionated plant and marine extracts (see supporting data). The screening was performed at a single concentration of 20.0 μM for purified natural products and sub-fractionated extracts were tested at 50.0 and 100 μg/mL. Biological activity data were normalized using the growth inhibition of pentamidine (positive control) at 120 nM and that of DMSO 0.5% (negative control) as the maximum and minimum values. The Z’ factor for the controls was calculated to be 0.83 using the equation defined as:

Z’ = 1 − 3(σ_C_^+^ + σ_C_^−^)/|μ_C_^+^ − μ_C_^−^|

where σ_C_^+^/σ_C_^−^ are the standard deviation (SD) of the positive/negative controls and μ_C_^+^/μ_C_^−^ are the mean values, indicating that the assay was performed with an excellent window between the two controls (Figure 2A). The results were plotted to evaluate the frequency distribution and a relatively high number of hits were identified from this screening (Figure 2B).

To compare the activity of our natural products to the control, the dose responsive *T.* *brucei* growth inhibition and cytotoxicity of pentamidine were also evaluated with an EC_50_ value of 0.015 ± 0.003 μM and CC_50_ < 0.40 μM. From the data, we have found that the extracts from Vietnamese marine invertebrates, the soft corals *L. crassum*,* L. laevigatum*,* S. dissecta* and the echinoderms *A. polyacanthus*, *D. Savignyi*, showed significant inhibitory effect against *T. brucei* (with EC_50_ values ranging from 0.36 ± 0.10 to 3.42 ± 0.32 μg/mL) and were therefore selected for a more detailed study of the activity of their chemical constituents.

### 2.2. Biological Activities of Isolated Constituents from the Selected Marine Organisms

The major constituents of the selected methanolic extracts with high activity were previously isolated in our laboratory (Y.H.K.). These compounds were laevigatol B (**1**) from *Lobophytum crassum* and *L. laevigatum* [14], (24*S*)-ergost-4-ene-3-one (**2**) from *Sinularia dissecta* [15], astropectenol A (**3**) and 5*α*-cholest-8(14)-ene-3*β*,7*α*-diol (**5**) from *Astropecten polyacanthus* [16] and cholest-8-ene-3*β*,5*α*,6*β*,7*α*-tetraol (**4**) from *Diadema savignyi* [17], respectively (Figure 1). Their structures were elucidated on the basis of spectroscopic data and comparison of them with reported values. For the present study, these compounds’ anti-trypanosomal activity against *T. brucei* as well as their cytotoxicity against human cell lines (HEK293T and HepG2) were determined. The data are reported in Table 1.

**Table 1:** *In vitro* antiparasitic and cytotoxic activities of marine metabolites.

Compounds	*T*.* brucei* EC_50_ Values (μM)	Cytotoxicity ^a^ CC_50_ Values (μM)		SI ^b^	Species	Ref.
		HEK293T	HepG2			
Laevigatol B (**1**)	5.34 ± 0.47	>100	>100	>18.7	*L. crassum* *L. laevigatum*	[14]
(24*S*)-Ergost-4-ene-3-one (**2**)	5.47 ± 0.27	>100	>100	>18.3	*S. dissecta*	[15]
Astropectenol A (**3**)	1.57 ± 0.14	>100	>100	>63.7	*A. polyacanthus*	[16]
Cholest-8-ene-3*β*,5*α*,6*β*,7*α*-tetraol (**4**)	14.60 ± 1.36	>100	>100	>6.8	*D. savignyi*	[17]
5*α*-Cholest-8(14)-ene-3*β*,7*α*-diol (**5**)	>30	>100	>100	-	*A. polyacanthus*	[16]
Pentamidine ^c^	0.015 ± 0.003	<0.40	<0.40	<26.7	-	-
Chlorpromazine ^c^	-	19.89 ± 3.48	16.50 ± 0.45	-	-	-

^a^ Selectivity relative to HEK293T and HepG2 cells for selected potent compounds; ^b^ SI: Selectivity index; ^c^ Positive control. All data represent the mean ± SD of at least three independent experiments performed in triplicates (*p* < 0.01).

**Figure 3:**
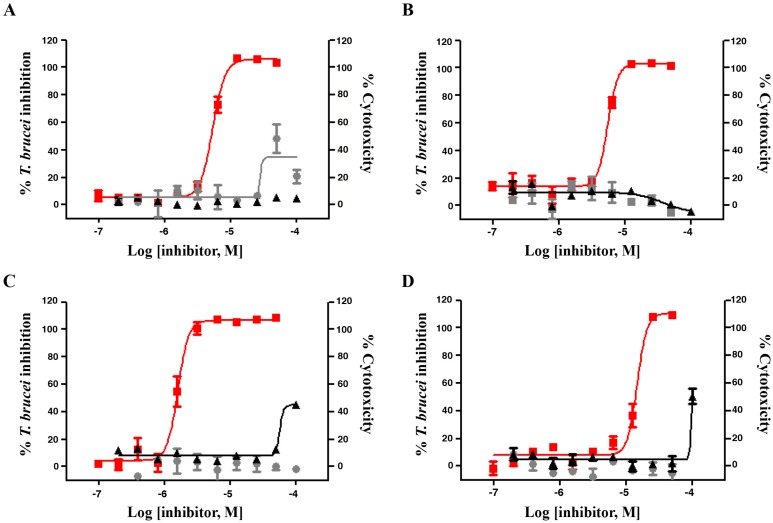
Inhibitory activity against *T*. *brucei* (red) and cytotoxic activity on HEK293T (grey) and HepG2 (black) cells of compounds **1** (**A**), **2** (**B**), **3** (**C**), and **4** (**D**).

Compounds **1**‒**4** displayed activity against *T. brucei* and no significant cytotoxicity against either tested cell line up to a concentration of 100 µM (the highest concentration tested). Only compound **5** did not show any significant anti-trypanosomal activity up to 30.0 µM. The anti-trypanosomal effect of compounds **1**‒**4** showed a clear dose-dependency (Figure 3) and all four compounds completely killed the parasites at a concentration of 20.0 µM. The EC_50_ concentrations were below 15.0 µM in all cases, astropectenol A (**3**) being the most active with an EC_50_ value of 1.57 ± 0.14 µM. Although its activity was about 100 times lower than that of the positive control (pentamidine) with an EC_50_ of only 0.015 ± 0.003 µM, it is worth noting that the selectivity index (SI) of **3** was markedly higher (63.7 *vs.* 26.7), so that this compound certainly represents an interesting starting point for further optimization. The relatively high SI value of compound **3** indicates that in this case anti-trypanosomal activity is based on a specific mechanism of action rather than on a general toxic effect.

Our previous work on the constituents of the soft corals *L. crassum* and *L. laevigatum* led to the identification of some novel cembranoid derivatives, including laevigatol B (**1**), which were evaluated for their anti-inflammatory and cytotoxic activities, as well as full assignment of all NMR data is reported [14]. In the current assay against *T*.* brucei*, laevigatol B (**1**) exhibited inhibitory activity with an EC_50_ value of 5.34 ± 0.47 μM (1.69 ± 0.12 µg/mL). Although its activity was considerably weaker than that of the positive control (pentamidine), laevigatol B (**1**) did not display cytotoxicity against both human cell lines, and thus showed a certain degree of selectivity relative (Table 1, Figure 3). However, it is interesting to note that the methanolic extract (EC_50_ = 0.36 ± 0.10 μg/mL) of this soft coral was more active than laevigatol B (**1**), suggesting the presence of other active derivatives, which could act individually or by synergy with laevigatol B (**1**). It is interesting to note in this respect that inhibition of parasites responsible for NTDs was already reported for cembrane-type diterpenes from soft coral species [18]. Similarly, Schmidt *et al.* reported that the cembranoid serratol obtained from the plant *Boswellia serrata* was active against *T. brucei rhodesiense* and *Plasmodium falciparum* (etiologic agent of tropical malaria) [19]. Further research on the antiprotozoal potential of cembrane diterpenes from marine or plant sources is thus warranted.

Comparing the observed effects between the structurally similar steroid derivatives (**2**‒**5**), (24*S*)-ergost-4-ene-3-one (**2**, EC_50_ = 5.47 ± 0.27 μM) and astropectenol A (**3**, EC_50_ = 1.17 ± 0.14 μM) showed stronger activity than that of cholest-8-ene-3*β*,5*α*,6*β*,7*α*-tetraol (**4**, EC_50_ = 14.6 ± 1.36 μM) and 5*α*-cholest-8(14)-ene-3*β*,7*α*-diol (**5**, EC_50_ > 30.0 μM). This observation suggested that the presence of a ketone and/or aldehyde group might play an important role in their anti-trypanosomal activities. This is in agreement with recent reports of the antiparasitic inhibitory activities of other steroids (such as pandaroside G methyl ester, norselic acid D, *etc.*) [20,21,22]. Our finding that compounds **1**‒**4** possess significant activity against *T. brucei* suggests that these compounds or related steroids might be interesting for further investigations for potential activity against related trypanosomatid human parasites.

**Figure 4:**
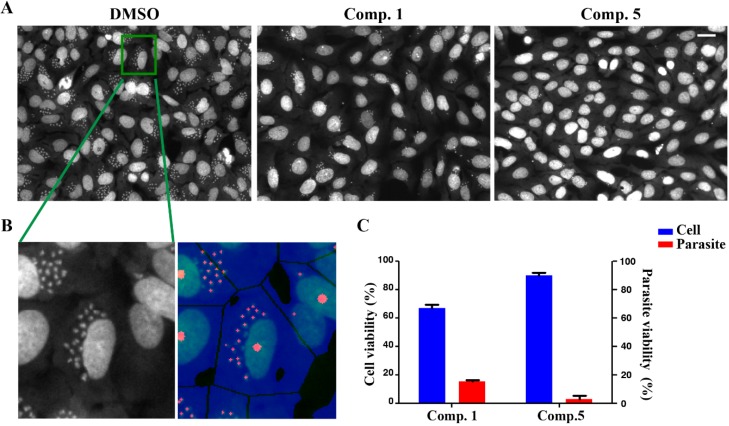
Assessment of the activity of laevigatol B (**1**) and 5*α*-cholest-8(14)-ene-3*β*,7*α*-diol (**5**) against intracellular amastigotes of *T. cruzi* by high content image assay coupled with quantitative image mining algorithm; (**A**) Images of intracellular *T. cruzi* with DMSO control (0.5%), compounds **1** and **5** at 50.0 μM; (**B**) Image analysis of acquired image. Large red dots indicates U2OS nuclei, blue surface refers to cytosol of the corresponding host cell and small red dots refers to *T. cruzi* nuclei; (**C**) Quantified cell and parasite numbers from acquired images (4 fields) of control, compounds **1** and **5** treat wells. Scale bar, 40.0 μM.

We therefore tested compounds **1**‒**5** also for growth inhibitory activity against the intracellular form of *T. cruzi*, the causative agent of Chagas’ disease (American trypanosomiasis). Quite interestingly, in this assay, only the cembranoid diterpene **1** (laevigatol B) and steroid **5** (5*α*-cholest-8(14)-ene-3*β*,7*α*-diol) showed significant activity. As shown in Figure 4A, integrity of the host cell is not compromised by treatment with compounds **1** and **5** in terms of cell shapes and number at a concentration of 50.0 μM whereas both lead to a significant reduction of the number of intracellular parasites (small dots) compared to dimethyl sulfoxide (DMSO) control. Interestingly, the steroid 5*α*-cholest-8(14)-ene-3*β*,7*α*-diol (**5**) which was inactive against *T. brucei*, showed superior activity. Compound **5** decreased parasite survival by 97.1%, compared to the DMSO control while the cell number of the host cells was not significantly reduced. The diterpene laevigatol B (**1**) showed reduction of parasites growth to 84.8%. However, **1** also caused a reduction of the host cells by 33%, comparable to the DMSO control (Figure 4C). Since compound **5** did not show any cytotoxicity to primary mammalian HEK293T and HepG2 cells at concentrations up to 100 μM (Table 1), it appears that its activity against intracellular forms of *T. cruzi* is based on a selective mechanism rather than on unspecific toxicity. Compounds **2**‒**4** did not exert significant inhibitory effects on this parasite (less than 30% inhibition at 50.0 µM).

To the best of our knowledge, no previous reports regarding the activity of the extracts and/or compounds derived from echinoderm species against parasites responsible for NTDs could be found [18].

## 3. Experimental

### 3.1. Biological Material

The samples of the soft corals (*Lobophytum crassum*, *L.* *laevigatum*, and *Sinularia dissecta*) and echinoderms (*Astropecten polyacanthus* and *Diadema savignyi*) were collected at Quangtri, Khanhhoa, Hue, and Haiphong, Vietnam, from April 2010 to May 2013, identified by Nguyen Thi My Ngan, M.Sc. (Institute of Oceanography) and Prof. Do Cong Thung (Institute of Marine Environment and Resources, VAST). A list of these samples can be found in earlier publications [14,15,16]. Voucher specimens were deposited at the Institute of Marine Biochemistry, Institute of Oceanography, and Institute of Marine Environment and Resources, VAST, Vietnam.

### 3.2. Chemicals, Reagents, and Media

Resazurin sodium salts, adenine, biotin, NaHCO_3_, phorbol 12-myristate 13-acetate, lectins PNA, paraformaldehyde (PFA) were purchased from Sigma (St. Louis, MO, USA); fetal bovine serum (FBS), HMI-9 medium, streptomycin/penicillin, HEPES, RPMI medium were purchased from Gibco (Grand Island, NY, USA); Draq5 was purchased from Biostatus (Leicestershire, UK); 384 well assay plates were purchased from Griener (Monroe, NC, USA).

### 3.3. Marine Product Derivatives

Each powdered material sample (150‒250 g) was extracted with MeOH (1 L) in glass jars (2 L) with screw-on lids. The methanolic extracts were filtered, combined, and concentrated using a rotary vacuum evaporator at a temperature about 40 °C and then further dried *in* *vacuo* at ambient temperature for 24 h. High purity compounds (>96%) were previously isolated and their structures elucidated from the methanolic extracts of the Vietnamese soft corals (*L. crassum*, *L. laevigatum*, and* S. dissecta*) and echinoderms (*A. polycanthus* and *D. savignyi*) [14,15,16,17]. Stock solutions of tested samples in DMSO were prepared, kept at −20 °C, and diluted to the final concentration in fresh media before each experiment. In order to ensure unaffected cell growth, the final DMSO concentration did not exceed 0.5% in all experiments.

### 3.4. Anti-Trypanosomal Activity on T. brucei

*T. brucei* strain 427 bloodstream forms were cultivated at 37 °C with 5% CO_2_ in HMI-9 medium supplemented with 10% FBS. Cultivated parasites (2,500) were seeded in 384 well plates with the natural compounds and incubated for 3 days. After the incubation, the parasites were exposed to 120 µM of resazurin sodium salt (10.0 µL of a 720 µM solution in water) for 5 h. Then, the parasites were fixed with 4% PFA and the assay plates were read by Victor 3™ (PerkinElmer, Waltham, MA, USA) at 530 nm_Ex_/590 nm_Em_. Pentamidine was used as a reference drug and DMSO 0.5% was used as a drug-negative control.

### 3.5. Anti-Trypanosomal Activity on T. cruzi Y Strain

The assay for activity against *T. cruzi* Y strain was performed as previously described [13]. Briefly*, T. cruzi* parasites were maintained in form of Tissue Culture Trypomastigote (TCT) by circulation in LLC-MK2 cell line (CCL-7, ATTC, Manassas, VA, USA). Seven days after the infection, the supernatant containing TCT parasites was collected and used to reinfect new cultures of LLC-MK2. The U2OS human cell (HTB-96, ATCC), cultivated in DMEM-high glucose media supplemented with 10% heat inactivated FBS, was used for the *in vitro* assay preparation. The cells were infected with the TCT form of *T. cruzi.* A homogeneous mixture of parasites and cells in DMEM-low glucose media, supplemented with 2% heat inactivated FBS, and was seeded into 384-well plates at 50.0 µL/well. The test compounds (final concentration of 50.0 µM) were added in 10.0 µL of 6% DMSO (final concentration, 1% DMSO) and the cells were incubated for 48 h at 37 °C, 5% CO_2_. After the incubation, the cells and parasites were fixed with 4% PFA and stained with 5.0 µM Draq5. Fluorescent images were acquired from each assay well using an Operetta microscope (Perkin Elmer).

### 3.6. Cytotoxicity Assays

HEK293T and HepG2 cells were cultured at 37 °C with 5% CO_2_ in Dulbecco’s modified eagle medium containing 10% FBS. Both cell types were plated individually in 384 well plates for testing the natural compounds at 3-fold dilution from 100 µM in 10-points concentration and incubated for 3 days. To determine viability, 10.0 µL of a 280 µM solution of resazurin sodium salt in water was added to the cells in the assay plate for at least 5 h (final concentration, 40.0 µM of resazurin) and resazurin reduction was measured with a Victor 3^TM^ fluorimeter (PerkinElmer) at 530 nm_Ex_/590 nm_Em_.

### 3.7. Statistical Analysis and Image Mining

The acquired images from the high content intracellular *T. cruzi* screening were subjected to in-house software to quantify cell numbers, parasite numbers, and infection ratio as previously described [13]. Each compound’s activity was normalized based on values of drug positive and negative control (120 nM of pentamidine for *T*. *brucei* and *T. cruzi* assays, 83.0 µM of chlorpromazine for cytotoxicity, and DMSO as 0% drug activity for negative control). Dose response curves were fitted by sigmoidal dose-response with variable slope, equation described as Y = Bottom + (Top − Bottom)/(1 + 10^(LogEC5 − X) − HillSlpe^) using GraphPad Prism 6 Software (GraphPad Software, Suite 230 La Jolla, CA, USA).

## 4. Conclusions

In conclusions, we have identified five inhibitors **1**‒**5** of trypanosomal growth from the marine soft corals and echinoderms. While compounds **1**‒**4** showed activity against bloodstream forms of *T*. *brucei*, only the cembranoid diterpene **1** and steroid **5** were active against intracellular amastigote forms of *T. cruzi*. Since none of the compounds displayed any significant cytotoxicity, these and related compounds may open a new avenue for future drug development against African sleeping sickness and/or Chagas’ disease. It should be noted at this point, that the strain of *T. brucei* studied here belongs to subsp. *brucei* which is not human pathogenic but responsible for trypanosomiasis in cattle. Further investigations with human pathogenic *T. brucei* (subsp. *gambiense* and/or *rhodesiense*) are therefore warranted. Subsequent studies will also include the assessment of underlying mechanisms of action of these compounds and evaluation of their *in vivo* efficacy in animal models, which could not be performed in the current study due to low available amounts of the isolated compounds.

## Supplementary Materials

Supplementary materials can be accessed at: http://www.mdpi.com/1420-3049/19/6/7869/s1.
