# Supplementary material for: Secondary Metabolites from Vietnamese Marine Invertebrates with Activity against Trypanosoma brucei and T. cruzi
Source: Molecules. 2014 Jun 11;19(6):7869–80. doi: 10.3390/molecules19067869 (PMC6271609; doi:10.3390/molecules19067869)
Supplement: Supplementary file 1 [file molecules-19-07869-s001.pdf]

Supplementary Material

Table S1. List of 87 hit samples inhibitory activity on *Trypanosoma brucei* growth.

No.	Description	Activity (%)	Cellratio (%)	MW	Note
1	**31A-PA-105A**	96.3	90.4	464	-
2	**31A-PA-89A**	96.2	91.1	356	-
3	**31B-AM-04G**	98.8	104.0	402	-
4	**31B-AM-05O**	91.2	104.2	380	-
5	**34A-AI-26B**	86.0	94.8	138	-
6	**34A-PM-129E**	83.4	102.7	426	-
7	**34A-PM-129F**	89.3	96.5	426	-
8	**34A-PM-129G**	85.2	100.5	428	-
9	**34A-PM-134D**	99.9	97.7	858	-
10	**34A-PM-81C**	100.7	74.8	-	extract
11	**34B-CS-40B**	98.6	106.4	426	-
12	**35A-LCR-12**	100.9	78.5	288	-
13	**35A-LCR-13**	101.6	75.3	318	-
14	**35A-LCR-2**	101.0	100.6	302	-
15	**35A-LCR-29**	103.8	97.0	352	-
16	**35A-SB-1**	86.9	104.3	402	-
17	**35A-SB-3**	100.4	90.9	402	-
18	**35A-SB-5**	100.0	92.3	418	-
19	**35A-SB-9**	101.1	83.3	432	-
20	**35A-SD-2**	102.5	91.7	398	-
21	**35A-SD-4**	105.1	100.8	465	-
22	**35A-SM-15**	103.7	100.3	322	-
23	**35A-SM-28**	101.9	74.6	432	-
24	**35B-DS-12**	106.3	80.2	386	-
25	**36A-CI-49M**	81.2	98.6	446	-
26	**36A-EH-19**	104.5	98.4	170	-
27	**36A-EH-26**	84.1	77.2	302	-
28	**36A-EH-30**	103.8	72.0	-	extract
29	**36A-EH-32**	103.3	89.9	-	extract
30	**36A-EH-6**	104.7	71.6	634	-
31	**39A-AK-33B**	100.3	100.8	308	-
32	**39A-PM-18F**	97.6	94.0	446	-
33	**KIH180**	88.9	93.8	410	-
34	**SpH1**	86.5	79.2	256	-
35	**SpH2**	86.6	90.2	312	-
36	**VHKC-0003**	101.2	96.6	-	extract
37	**VHKC-0005**	101.2	92.1	-	extract
38	**VHKC-0014**	98.8	78.4	-	extract
39	**VHKC-0048**	100.8	72.5	-	extract
40	**VHKC-0058**	84.6	73.5	-	extract
41	**VHKC-0062**	101.6	92.0	-	extract
42	**VHKC-0064**	98.2	85.9	-	extract
43	**VHKC-0072**	98.7	106.4	-	extract

Table S1. *Cont.*

No.	Description	Activity (%)	Cellratio (%)	MW	Note
44	**VHKC-0082**	99.8	84.2	-	extract
45	**VHKC-0083**	101.1	76.5	-	extract
46	**VHKC-0084**	101.2	75.9	-	extract
47	**VHKC-0108**	98.9	99.3	-	extract
48	**VHKC-0113**	85.8	90.0	-	extract
49	**VHKC-0123**	101.7	78.8	-	extract
50	**VHKC-0128**	97.4	96.6	-	extract
51	**VHKC-0129**	101.4	105.5	-	extract
52	**VHKC-0131**	99.8	72.4	-	extract
53	**VHKC-0139**	86.4	78.4	-	extract
54	**VHKC-0142**	101.0	80.7	-	extract
55	**VHKC-0147**	101.8	105.6	-	extract
56	**VHKC-0191**	101.6	78.3	-	extract
57	**VHKC-0201**	91.6	79.5	-	extract
58	**VHKC-0208**	102.9	80.9	-	extract
59	**VHKC-0212**	87.7	91.0	-	extract
60	**VHKC-0216**	101.4	76.5	-	extract
61	**VHKC-0219**	88.5	75.4	-	extract
62	**VHKC-0232**	104.2	90.2	-	extract
63	**VHKC-0240**	82.8	105.3	-	extract
64	**VHKC-0247**	104.6	81.1		extract
65	**VHKC-0249**	104.2	71.1	-	extract
66	**VHKC-0259**	101.8	85.8	-	extract
67	**VHKC-0263**	103.8	72.8	-	extract
68	**VHKC-0265**	104.4	74.5	-	extract
69	**VHKC-0271**	103.1	76.8	-	extract
70	**VHKC-0273**	80.9	102.9	-	extract
71	**VHKC-0295**	104.6	83.0	-	extract
72	**VHKC-0306**	102.7	80.0	-	extract
73	**VHKC-0332**	95.5	84.5	-	extract
74	**VHKC-0344**	103.0	83.7	-	extract
75	**VHKC-0354**	103.0	88.3	-	extract
76	**VHKC-0403**	95.6	81.2	-	extract
77	**VHKC-0417**	103.3	81.1	-	extract
78	**VHKC-0429**	92.2	70.9	-	extract
79	**VHKC-1051**	85.5	96.7	-	extract
80	**VHKC-1052**	98.1	91.8	-	extract
81	**VHKC-1053**	83.9	102.0	-	extract
82	**VHKC-1082**	85.6	73.5	-	extract
83	**VHKC-1089**	104.5	83.4	-	extract
84	**VHKC-1090**	95.0	87.0	-	extract
85	**VHKC-1096**	80.7	94.2	-	extract
86	**VHKC-5057**	103.6	84.7	-	extract
87	**VHKC-5060**	103.9	87.4	-	extract
